# Key Components of Cognitive Remediation for Schizophrenia: A Bayesian Network Meta-Analysis

**DOI:** 10.1192/j.eurpsy.2025.561

**Published:** 2025-08-26

**Authors:** L. Van Der Meer, T. Brands, T. van Brouwershaven, M. L. Thomas, N. Buist, E. Twamley, A. Vita, G. H. Pijnenborg, A. Poppe

**Affiliations:** 1 Clinical & Developmental neuropsychology, University of Groningen, Groningen; 2 Department of Rehabilitation, Lentis Psychiatric Institute, Zuidlaren, Netherlands; 3 LVR Viersen, Viersen, Germany; 4 Department of Psychology, Colorado State University, Fort Collins; 5 Department of Psychiatry, University of California San Diego; 6 Center of Excellence for Stress and Mental Health, VA San Diego Healthcare System, San Diego, United States; 7 Department of Clinical and Experimental Sciences, University of Brescia, Brescia, Italy; 8 Department of Psychotic Disorders, GGZ Drenthe, Assen; 9 Radboud university, Nijmegen, Netherlands

## Abstract

**Introduction:**

Cognitive impairments are strongly associated with impaired everyday functioning in individuals with schizophrenia, and cognitive remediation (CR) has been identified as effective treatment. However, uncertainty remains regarding the most effective components of CR.

**Objectives:**

To identify (1) the most effective combination of core ingredients of CR (cognitive exercises, presence of a therapist, cognitive strategies, and generalization activities) and (2) the most effective type of CR to improve everyday functioning in individuals with schizophrenia (computer-assisted cognitive remediation, single domain computer-assisted cognitive remediation, social cognition interventions, paper and pencil interventions, integrative approaches, and combination approaches).

**Methods:**

PubMed, PsycInfo, Medline, Embase were searched literature from inception until November 25, 2022. Reference lists of included studies and previous meta-analyses were searched for relevant studies. We included randomized controlled trials comparing CR with any control condition or a different type of CR, assessing everyday functioning pre- and post-intervention in individuals with schizophrenia. The studies were selected independently by two reviewers. We followed the PRISMA guidelines. Study data were extracted independently by two reviewers. Data were analyzed using Bayesian random-effects network models. Trial methodological quality was evaluated with the Clinical Trials Assessment Measure. Risk of bias was evaluated. Primary outcomes were changes in functioning and cognition from baseline to after CR and from baseline to followup (min. 3 months). Literature search was updated in September 2024.

**Results:**

86 studies and 6076 participants were included. For both outcomes, the most effective constellation of core elements of CR were cognitive exercises, CR provided by a therapist and the use of generalization procedures (functioning: g = 0.31, 95% CrI [0.14, 0.47]; cognition: g = -0.23, 95% CrI [0.03, 0.42]). Moreover, only combinations that included both cognitive exercises and a therapist were more effective than TAU. All four core elements were necessary to observe improved functioning at follow-up. For the specific types of CR, none was superior to any other CR type. All CR types were more effective in improving functioning compared to TAU (g = 0.19-0.45). Study quality did not influence the results. Results will be updated if necessary after inclusion of the updated literature search.

**Conclusions:**

The findings indicate that the effectiveness of CR depends on the inclusion of four essential elements—cognitive exercises, therapist involvement, strategies, and generalization activities—rather than on the specific type of CR intervention. An update to the definition of CR is recommended.

**Disclosure of Interest:**

None Declared

